# Outcomes of Endoscopic Stapedectomy: Systematic Review

**DOI:** 10.1055/s-0043-1761171

**Published:** 2024-02-05

**Authors:** Ahmed Nabil Elsamnody, Amr Hamdino Yousef, Mohamed Salah Taha

**Affiliations:** 1 Department of Otorhinolaryngology, Al-Azhar University Hospitals, Cairo, Egypt

**Keywords:** endoscopy, conductive hearing loss, otosclerosis, tinnitus

## Abstract

**Introduction** Stapes surgery was traditionally performed with the use of microscopy either through postauricular, endaural or transcanal approaches. Endoscopic stapedectomy ushered a revolution as a new technique with less complications.

**Objective** To review the outcomes of endoscopic stapes surgery with an emphasis on intraoperative and postoperative clinical and audiological results.

**Data Synthesis** A literature review on the PubMed, Web of Science, Scopus, the Cochrane Library, and Embase databases was conducted. *Endoscopic stapes surgery* or *stapedotomy* were the main keywords used, and we searched for studies and research published from January 2015 to October 2021. Articles on endoscopic stapes surgery were included, and qualitative and descriptive analyses of the studies and outcomes data regarding audiometric changes and postoperative complications were conducted. Articles including patients with cholesteatoma were excluded. A total of 122 studies were retrieved for qualitative and descriptive analyses and to measure the outcomes of endoscopic stapedotomy; only 12 studies met the inclusion criteria, and the rest was excluded. The meta-analysis revealed a statistically significant difference in hearing improvement. The gain in air-bone gap ranged from 9 dB to 16 dB. A low rate of operative and postoperative complications was reported.

**Conclusions** Endoscopic stapes surgery appears to be a reasonable alternative to microscopic stapes surgery, with shorter operative times, low complication rate, and significant hearing improvement. The endoscopic technique enabled a better visualization and less scutum drilling, which was confirmed by all included studies.

## Introduction

Stapes surgery was traditionally performed with the use of microscopy, either through postauricular, endaural or transcanal approaches. Anatomical variations in the external auditory canal, such as abnormal bony hump or narrow canal, are considered hindering factors for the transcanal or endaural approaches. 1 Stapedotomy with a postauricular incision has many complications, such as bad cosmesis, pain, auricular numbness, and postoperative infection. 2

Otologic microscopic surgery has many advantages, such as good magnification, visibility, and the perception of depth. Microscopic surgery enables otologists to use both hands (two-handed technique). 2

Initially, rigid endoscopes were used in ear surgery as an adjunct to microscopes for diagnostic purposes, whereas the use of endoscopes in operative approaches was first described with Peo DSin 2000. To provide better outcomes for the patients, including audiological improvement and minimal postoperative morbidity, these approaches require more training on the apert of the surgeons. Endoscopic ear surgery enables a better visualization of middle ear mucosal folds and deep recesses of the middle ear to better detect any pathology, such as residual cholesteatoma, or variations in the ventilation system of the middle ear. 3

Endoscopic otologic surgery has been increasingly applied in the surgical treatment of otosclerosis, with potential advantages over standard microscopic surgery. Several studies 4 5 have mentioned better visualization, lower chance of damaging periauricular structures, lower chance of chorda tympani injury, and minimal scutum drilling, with low postoperative complications, such as changes in the sense of taste, auricular numbness, pain, and short operative time.

The main objective of the present study is to compare the operative and postoperative clinical and audiological results and to review the outcomes and complications of endoscopic stapes surgery.

## Review of the Literature

**Fig. 1:**
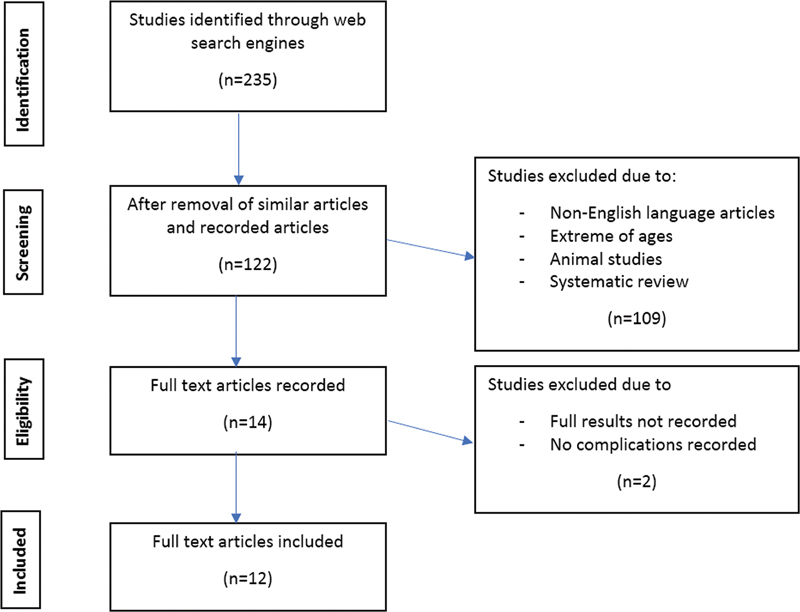
PRISMA flow chart of the present study.

A systematic review of the literature on endoscopic stapes surgery was performed according to the Preferred Reporting Items for Systematic Reviews and Meta-Analysis (PRISMA) statement. 6 We conducted a comprehensive search on the PubMed, Web of Science, Scopus, the Cochrane Library, and Embase databases. Endoscopic stapes surgery, or stapes fixation, or stapes prothesis, or stapedectomy, or stapedotomy were the main keywords used in the search for studies and research published from January 2015 to October 2021. Studies involving endoscopy stapes surgery, ossicular chain malformation or stapes malformation were included. The authors searched the literature independently and compared results at each stage of the PRISMA flow chart ( Fig. 1 ).

Studies evaluating operative techniques, audiometric changes, and postoperative complications of endoscopic stapes surgery in adult patients (aged between 18 and 45 years) were included. The exclusion criteria were articles not published in English, papers including patients with extreme of ages, conference proceedings, animal studies, uncompleted full-text papers, and articles on patients with associated pathologies, such as cholesteatoma.

All statistical analyses were performed with a 95% confidence interval (95%CI), and values of *p* < 0.05 were considered statistically signiﬁcant. For the analysis of the data, we used the Statistical Package for the Social Sciences (SPSS, SPSS Inc., Chicago, IL, United States) software, version 15.0. In addition to the standard descriptive statistical calculations, such as mean, standard deviation (SD), the results of the categorical variables were presented as numbers and percentages.

Initially, 235 studies were identified ( Fig. 1 ); with the removal of similar articles, 122 studies remained. After the application of the exclusion criteria, 108 articles were excluded. After a full-text review, only 12 articles published between January 2015 and October 2021 were included in the present systematic review.

The mean age of the 371 patients who composed the total sample of the studies included was of 40.3 years old. The plastic prothesis was used in 5 studies (in a total of 95 patients), while the Teflon prothesis was used in 4 studies (in a total of 100 patients). Only 2 studies used the titanium prothesis (in a total of 168 patients), and in 1 study, the 8 patients involved received a hydroxyapatite prothesis ( Table 1 ).

**Table 1:** Data on the studies included in the systematic review

Author (year of publication)	No. of patients	Mean age of the patients (years)	Gender (male/female)	Side of affected ear (right/left)	Follow-up (months)	Type of prothesis
Daneshi and Jahandideh 7 (2016)	19	36.7	7/12	–	7.42	Plastic
Ianella and Magliulo 8 (2016)	19	44.3	7/13	9/11	10.3	Plastic
Dursun et al. 9 (2016)	31	41.5	13/18	–	6	Plastic
Naik and Nemade 16 (2016)	20	32.7	13/7	–	1.5	Plastic
Marchioni et al. 10 (2016)	6	34	3/3	3/3	36	Plastic
Sproat et al. 11 (2017)	34	47	20/14	15/19	5	Teflon
Bhardwaj et al. 12 (2018)	20	33	12/8	–	6	Teflon
Plodpai et al. 2 (2017)	18	38	15/3	9/9	6	Titanium
Monier et al. 13 (2017)	14	33.6	–	–	4.5	Teflon
Gulsun et al. 3 (2019)	32	33	17/15	20/18	6	Teflon
Bianconi et al. 15 (2020)	150	48.2	66/84	90/60	4	Titanium
Hosoya et al. 17 (2021)	8	61.6	1/7	–	12	Hydroxyapatite

**Fig. 2:**
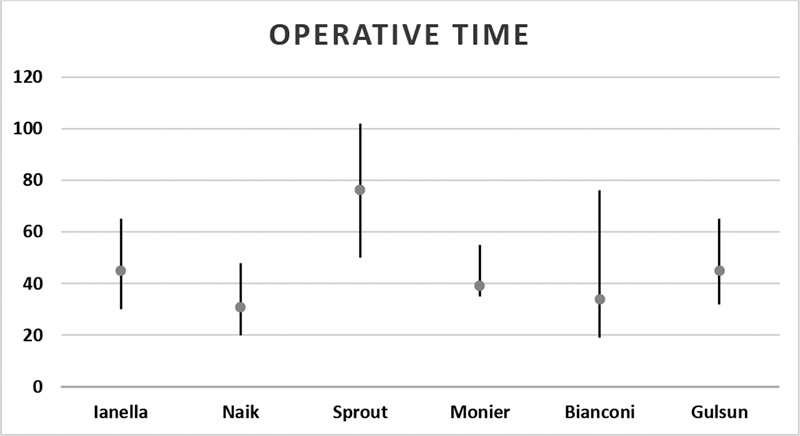
Operative times of the included studies.

Closure of the air-bone gap (ABG) was less than 20 dB in 347 cases of 12 studies while only 281 patients had ABG closure less than 10 dB ( Table 2 ). In 6 studies, the mean operative time was of 45.5 minutes, ranging from 102 to 19 minutes ( Fig. 2 ).

**Table 2:** Air-bone gap closure to > 20 dB and > 10 dB

Author (year of publication)	No. of patients	Air-bone gap closure to > 20 dB (n)	Air-bone gap closure to > 10 dB (n)
Daneshi and Jahandideh 7 (2016)	19	17	11
Ianella and Magliulo 8 (2016)	19	19	17
Dursun et al. 9 (2016)	31	30	19
Naik and Nemade 16 (2016)	20	20	20
Marchioni et al. 10 (2016)	6	6	4
Sproat et al. 11 (2017)	34	29	27
Bhardwaj et al. 12 (2018)	20	18	–
Plodpai et al. 2 (2017)	18	15	15
Monier et al. 13 (2017)	14	13	10
Gulsun et al. 3 (2019)	38	33	32
Bianconi et al. 15 (2020)	150	139	118
Hosoya et al. 17 (2021)	8	8	8

Among the included studies, there were: only 6 cases of postoperative hearing loss due to injury to the stapes footplate and underlying oval window; 31 cases of postoperative dizziness lasting more than 1 day after the operation; only 24 cases of injury to the chorda tympani that presented with postoperative dysgeusia; and only 2 cases of postoperative transient facial palsy ( Table 3 ).

**Table 3:** Complications reported by the studies included

Author (year of publication)	Sensorineural hearing loss (n)	Dizziness (n)	Chorda injury (n)
Daneshi and Jahandideh 7 (2016)	0	2	0
Ianella and Magliulo 8 (2016)	0	4	0
Dursun et al. 9 (2016)	0	0	5
Naik and Nemade 16 (2016)	0	0	0
Marchioni et al. 10 (2016)	0	4	4
Sproat et al. 11 (2017)	0	2	2
Bhardwaj et al. 12 (2018)	0	3	0
Plodpai et al. 2 (2017)	0	1	0
Monier et al. 13 (2017)	0	1	1
Gulsun et al. 3 (2019)	1	1	7
Bianconi et al. 15 (2020)	1	9	1
Hosoya et al. 17 (2021)	0	4	4

## Discussion

The better intraoperative view of the middle ear anatomical structures, particularly the stapes footplate, represents an advantage of the endoscopic approach during stapes surgery. 7

There are many challenges for endoscopic stapes surgery. Studies mention loss of depth perception and potential difficulties in prosthesis manipulation associated with a single-handed insertion technique. The better audiological gain and closure of the ABG and the lower rate of complications are the main factors that motivate otologists to choose their preferred technique. 8

In the studies included, the rate of ABG closure less than 20 dB for endoscopic stapedotomy was of 94.04%, and that of ABG closure less than 10 dB was of 76.2%. The ABG closure ranged from 100% to 90.63% less than 20 dB and from 100% to 78.67% less than 10 dB. 8 9 10 11 12 13 14 15 This could be explained because, in some of the included studies in which the mean age of the patients was > 45 years, such as those by Sproat et al. 11 (2017) and Bianconi et al. 15 (2020), the rate of ABG closure less than 20 dB was 90.63% and 92.67% respectively, and the rate of ABG closure less than 10 dB was 84.38% and 78.67% respectively.

The mean operative time varied among the studies, which could be explained by the availability of the appropriate equipment or the experience of the surgeons. In the study by Iannella and Magliulo, 8 the mean operative time was of 45 (range: 35 to 55) minutes; in Naik and Nemade, 16 it was of 31 (range: 20 to 48) minutes; in Sproat et al., 11 it was of 76 (range: 50 to 102) minutes; in Monier et al., 13 39 (range: 35 to 55)minutes; in Gulsen and Karatas, 14 45 (range: 32 to 65) minutes; and in Bianconi et al., 15 it was of 34 (range: 19 to 76) minutes, for example. Considering these studies, 8 11 13 14 15 16 the overall mean operative time was of 45.5 (range: 102 to 19) minutes.

Postoperative complications after stapes surgery vary from sensorineural hearing loss (SNHL) and dizziness to dysgeusia due to injury to the chorda tympani. Postoperative SNHL was only observed in 1.66% (361 patients) of the cases of the present review. There were two cases of hearing impairment immediately after the operation, one each in the studies by Gulsen and Karatas 14 and Bianconi et al., 15 representing rates of 3.125% and 0.67% respectively.

Regarding transient postoperative dizziness, the overall rate was of 8.4% (range: 2.63% to 66.67%) cases. Marchioni et al. 10 reported the highest rate, of 66.67%, followed by Hosoya et al. 17 (50%), while Gulsen and Karatas 14 reported the lowest rate: 2.63%. Dursun et al. 9 and Naik and Nemade 16 did not report cases of postoperative dizziness. Poldpai et al., 2 Gulsen and Karatas 14 and Monier et al. 13 each observed only one case of postoperative dizziness (with rates of 5.56%, 7.14%, and 2.63% respectively), and Bianconi et al. 15 found a rate of 6%. The tip of the endoscope induces thermal injury to the inner ear; this is the main explanation for the postoperative dizziness and hearing affection observed in certain studies. 2 9 10 13 14 15 16 17

Dysgeusia following stapes surgery can occur even with preservation of the chorda tympani, and the overall rate in the present systematic review was of 6.5% Marchioni et al. 10 reported the highest rate (4 out of 6 cases), followed by Hosoya et al. 17 There were no recorded cases in the studies by Daneshi and Jahandideh, 7 Ianella and Magliulo, 8 Naik and Nemade, 16 Bhardwaj et al., 12 and Plodpai et al. 2 Monier et al. 13 and Bianconi et al. 15 found only one case each, and the ratesin the studies by Dursun et al. 9 and Gulsen and Karatas 14 were of 16.13% and 18.42% respectively. Marchioni et al. 10 only included stapes malformation, which explains the high rate of complications observed in their study. But the other articles 2 7 8 9 10 11 12 13 14 15 16 17 reported far less complications in ears with normal anatomy.

Based on the analysis of the overall data, endoscopic stapedotomy seems to be safer than the microscopic technique. An important advantage of the endoscopic approach is the possibility of managing ossicular chain malformation, stapes malformation, and facial nerve dehiscence due to the better visualization. This is especially with experienced surgeon who had well trained for endoscopic manipulation of the middle ear structures and stapes prosthesis.

## Conclusion

Endoscopic stapedotomy is a technique with many advantages, such as good magnification, visibility, and depth perception. Although requires more training to manipulate the delicate structures of the middle ear, there is a lower chance of complications such as injury to the inner ear or chorda tympani. A comparison of the pros and cons of the endoscopic and microscopic approaches was not performed in the present meta-analysis. Although microscopic ear surgery is still preferred to stapedectomy, endoscopies are being increasingly used worldwide.
